# The Legacy of Parker, Baker and Smith 1972: Gamete Competition, the Evolution of Anisogamy, and Model Robustness

**DOI:** 10.3390/cells10030573

**Published:** 2021-03-05

**Authors:** Jussi Lehtonen

**Affiliations:** Faculty of Science, School of Life and Environmental Sciences, The University of Sydney, Sydney, NSW 2006, Australia; jussi.lehtonen@iki.fi

**Keywords:** isogamy, anisogamy, sexes, female, male, gamete, egg, sperm, reproduction, game theory

## Abstract

The evolution of anisogamy or gamete size dimorphism is a fundamental transition in evolutionary history, and it is the origin of the female and male sexes. Although mathematical models attempting to explain this transition have been published as early as 1932, the 1972 model of Parker, Baker, and Smith is considered to be the first explanation for the evolution of anisogamy that is consistent with modern evolutionary theory. The central idea of the model is ingenious in its simplicity: selection simultaneously favours large gametes for zygote provisioning, and small gametes for numerical competition, and under certain conditions the outcome is anisogamy. In this article, I derive novel analytical solutions to a 2002 game theoretical update of the 1972 anisogamy model, and use these solutions to examine its robustness to variation in its central assumptions. Combining new results with those from earlier papers, I find that the model is quite robust to variation in its central components. This kind of robustness is crucially important in a model for an early evolutionary transition where we may only have an approximate understanding of constraints that the different parts of the model must obey.

## 1. Introduction

In 1972 Geoff Parker, Robin Baker and Vic Smith [1] published a theoretical explanation for the evolution of anisogamy (see Table 1 for definitions), thus explaining why so much of life is organized around a reproductive system with gametes of two different sizes such as eggs and sperm. Furthermore, given that the biological definition of the sexes is based on a difference in gamete size, the theory is also an explanation for the evolutionary origin of females and males in an ancestral, broadcast spawning population. This transition has major consequences for subsequent evolution, and is a part of the so-called ‘sexual cascade’ [2] where the asymmetry in gamete size is thought to play a central role in driving sex-specific selection [3,4,5,6]. While Parker, Baker and Smith’s model [1] (henceforth, the ‘PBS’ model, as it has come to be known) was not the first model of the evolution of anisogamy, it was the first to provide an individual selection (as opposed to group selection) explanation [7,8], and hence the first one that is consistent with the logic of evolutionary theory. Like many of the best ideas, the theory has an air of ingenious simplicity about it, encapsulated in just a few lines on p. 531 of the 1972 paper [1]: “*Two very fundamental pressures immediately appear obvious; both would be related to gamete size and would act in opposition. These are numerical productivity (i.e., the number of gametes produced in unit time by a given parent) and zygote fitness (i.e., a measure of the probability that a zygote will survive to reach adulthood and reproduce, and in the shortest time)*”. In other words: there are simultaneous selection pressures for both large (for zygote provisioning) and small (for competition in numbers) gametes, and under certain conditions the outcome is anisogamy.

The second article celebrated alongside PBS in this theme issue, the 1970 “Sperm competition and its evolutionary consequences in the insects” [9] is the one that has gained more attention and citations out of the two. Perhaps this is partly due to the more immediate empirical applicability and testability of sperm competition theory in contemporary organisms, whereas the 1972 article [1] concerns an ancestral transition that takes place over evolutionary timescales, is not directly observable, and is difficult to test. The two articles are in my view of equal importance, and it may not be fruitful to compare them in terms of citation numbers or other typical metrics. They are also linked to each other in a chicken and egg -like manner: sperm competition in its usual form is a consequence of gamete dimorphism, where the smaller gamete type seemingly competes for the larger one. However, the evolution of gamete dimorphism is itself dependent on a precursor of sperm competition. As sperm did not exist before anisogamy evolved, I instead use the term gamete competition, where smaller gametes are potentially advantageous because they are more numerous in competition with other gametes. The two articles are therefore intimately linked, both in their logic and in their history. Their history is detailed elsewhere in this issue by its honouree, Geoff Parker [7]. In this article I focus on some theoretical and mathematical aspects of the topic.

There have been several subsequent re-analyses and extensions of the ideas introduced by PBS [1], making use of alternative methodologies including simulations [1,10], population genetics [11,12], and game theory [13,14,15,16,17,18]. I will discuss and build upon the work of Bulmer and Parker [15], which is a game-theoretical update to the PBS theory and provides a clear foundation for new analyses, generalisations, and for drawing connections to other models. I will begin by deriving a novel analytical solution to the main model of Bulmer and Parker [15]. This analytical solution provides further insight into the results of the model and addresses related misconceptions. I will then discuss and analyse the robustness of the model to variation in its central assumptions and components. Finally, I will discuss the connection of the Bulmer and Parker [15] model to so-called ‘gamete limitation’ models for the evolution of anisogamy.

## 2. Materials, Methods, and Results

### 2.1. A New Analytical Solution to Bulmer & Parker’s Game-Theoretical Model

Bulmer and Parker [15] presented a series of game-theoretical models of the evolution of anisogamy, partly motivated by criticisms aimed at the PBS theory and its assumptions [19,20,21] (see also [7] in this issue), and partly inspired by the game-theoretical models of Maynard Smith [13,14]. For their main model Bulmer and Parker [15] present an approximate solution for the anisogamous equilibrium and an exact analytical solution for the isogamous equilibrium. Here I build on their work by deriving an analytical solution to the model for all equilibria. This new solution has multiple advantages: it is exact rather than approximate, deepens insight into the model results, and clarifies a misunderstanding that has arisen from the original, numerical solution. Furthermore, as we will see in the next section, the analytical solution has a natural generalisation which confirms Parker’s (ref. [22], p. 36) conjecture that the overall results are not dependent on an exact trade-off between gamete size and number.

I begin with equations (2.1a–2.1b) from Bulmer and Parker [15], using a slightly modified notation where I denote gamete sizes of two mating types by *x* and *y* (instead of $m_{1}$ and $m_{2}$ as used by Bulmer and Parker). The fitness functions for the two mating types are

(1) $$\left\{\begin{array}{c} w_{x}\left(x,y\right)=n\left(x\right)g\left(x\right)f\left(x,y\right)\\ w_{y}\left(x,y\right)=n\left(y\right)g\left(y\right)f\left(x,y\right) \end{array}\right.$$

where *n*, *g*, and *f* are functions for gamete size-number trade-off, gamete survival, and zygote survival, respectively. In other words, the function *n* accounts for the selection pressure of numerical productivity and *f* for the selective pressure of zygote fitness described on p. 531 of PBS [1] quoted in the introduction. Using gamete size for mating type *x* as an example, these functions are $n\left(x\right)=M/x$, $g\left(x\right)=e^{-\alpha /x}$ and $f\left(x,y\right)=e^{-\beta /\left(x+y\right)}$ where *M* is the total resource budget for gamete production and α and β are positive parameters scaling the size-dependence of gamete and zygote survival, respectively [15]. Under these assumptions the logarithm of fitness is

(2) $$\left\{ \begin{array}{c} \ln w_{x}=\ln\left(\frac{M}{x}\right)-\frac{\alpha }{x}-\frac{\beta }{x+y}=\ln\left(M\right)-\ln\left(x\right)-\frac{\alpha }{x}-\frac{\beta }{x+y}\\ \ln w_{y}=\ln\left(\frac{M}{y}\right)-\frac{\alpha }{y}-\frac{\beta }{x+y}=\ln\left(M\right)-\ln\left(y\right)-\frac{\alpha }{y}-\frac{\beta }{x+y} \end{array}\right.$$

The direction of selection can then be calculated by differentiating logarithmic fitness for *x* and *y*, respectively, and at equilibrium these derivatives must vanish [15]:

(3) $$\left\{\begin{array}{c} \frac{\partial \ln w_{x}}{\partial x}=\frac{\alpha }{x^{2}}+\frac{\beta }{{\left(x+y\right)}^{2}}-\frac{1}{x}=0\\ \frac{\partial \ln w_{y}}{\partial y}=\frac{\alpha }{y^{2}}+\frac{\beta }{{\left(x+y\right)}^{2}}-\frac{1}{y}=0 \end{array}\right.$$

Note that instead of differentiating log fitness as above, we could equally well differentiate absolute fitness ($\frac{\partial w_{x}}{\partial x}$) and none of the resulting equilibria would be affected (as in, e.g., [18] where equilibria as well as the approximations of evolutionary trajectories are unaffected by this choice). Differentiating log fitness (as above and in Bulmer and Parker [15]) is equivalent to first differentiating fitness and subsequently dividing the derivative by fitness $\left(\frac{\partial \ln w_{x}}{\partial x}=\frac{\partial w_{x}}{w_{x}\partial x}\right)$, a consequence of the rules of differentiation. Any of these alternatives can be used if we are only interested in the direction of evolutionary change and in evolutionary equilibria. If the aim is also to estimate the magnitude of evolutionary change (e.g., over one generation) we must use $\frac{\partial \ln w_{x}}{\partial x}$ or $\frac{\partial w_{x}}{w_{x}\partial x}$ [23]. In short, although the choice does not matter in the models of this article and in many other applications, it is important to be aware that under some circumstances the difference is important.

Here I depart from the derivation of Bulmer and Parker [15]. Multiplying the top and bottom Equation (3) by $x^{2}$ and $y^{2}$, respectively, obtains

(4) $$\left\{\begin{array}{c} \alpha +\frac{\beta x^{2}}{{\left(x+y\right)}^{2}}-x=0\\ \alpha +\frac{\beta y^{2}}{{\left(x+y\right)}^{2}}-y=0 \end{array}\right.$$

Subtracting the bottom equation in (4) from the top one, we have

(5) $$\frac{\beta \left(x^{2}-y^{2}\right)}{{\left(x+y\right)}^{2}}-\left(x-y\right)=\left(x-y\right)\left[\frac{\beta }{\left(x+y\right)}-1\right]=0$$

Equation (5) has two alternative solutions: x=y or x+y=β. The former corresponds to isogamy, while the latter is a constraint on the anisogamous solution, but neither is yet the final solution for actual gamete sizes. The full solutions can be found by substituting these intermediate solutions back into Equation (4):

For the isogamous solution, first substitute x=y:

(6) $$\alpha +\frac{\beta x^{2}}{{\left(x+y\right)}^{2}}-x=0=\alpha +\frac{\beta x^{2}}{{\left(2x\right)}^{2}}-x=\alpha +\frac{\beta }{4}-x=0$$

which has the solution

(7) $$x=y=\alpha +\frac{\beta }{4}$$

Next, to find the anisogamous solutions substitute x+y=β:

(8) $$\alpha +\frac{\beta x^{2}}{{\left(x+y\right)}^{2}}-x=\alpha +\frac{\beta x^{2}}{{\left(\beta \right)}^{2}}-x=\alpha +\frac{x^{2}}{\beta }-x=0$$

In other words, finding the anisogamous gamete sizes amounts to solving the standard form quadratic equation $\frac{x^{2}}{\beta }-x+\alpha =0$ using the quadratic formula:

(9) $$x=\frac{1\pm \sqrt{1-4\frac{\alpha }{\beta }}}{2\left(\frac{1}{\beta }\right)\;}=\frac{\beta }{2}\left(1\pm \sqrt{1-\frac{4\alpha }{\beta }}\right)$$

Whichever solution (+ or −) of Equation (9) we choose for *x*, subsequently solving the equation x+y=β yields the other solution for y. The biological interpretation is that the two alternative solutions indicated by + and − in the quadratic formula correspond to the sizes of the macro- and microgametes, respectively, and we can arbitrarily assign one of them to x and the other to y.

Bulmer and Parker [15] show that the isogamous solution is stable when β<4α, and is replaced by the anisogamous solution when β>4α. Accordingly, Equation (9) only takes on real values when β≥4α, and is identical in value with the isogamous solution when β=4α. Therefore, the full set of stable equilibria can be characterised as follows:

(10) $$\left\{\begin{array}{c} \left\{x,y\right\}=\left\{\left(\alpha +\frac{\beta }{4}\right)\;,\;\;\left(\alpha +\frac{\beta }{4}\right)\right\}\;\;\;\;\;\;\;\;\;\;\;\;\;\;\;\;\;\;\;\;\;\;\;\;\;\;\;\;\;\;\;\;\;\;\;\;\;\;\;\;\;\;\beta \leq 4\alpha \;\\ \left\{x,y\right\}=\left\{\frac{\beta }{2}\left(1+\sqrt{1-\frac{4\alpha }{\beta }}\right)\;,\;\;\frac{\beta }{2}\left(1-\sqrt{1-\frac{4\alpha }{\beta }}\right)\right\}\;\;\;\;\;\;\;\;\;\;\;\;\beta >4\alpha \end{array}\right.$$

where *x* has been arbitrarily chosen to represent macrogamete size and *y* to represent microgamete size. The approximate result for anisogamy presented by Bulmer and Parker [15] can be recovered with a first order Taylor polynomial [24] for the square root (binomial approximation): $\sqrt{1-\frac{4\alpha }{\beta }}\approx 1-\frac{1}{2}\frac{4\alpha }{\beta }=1-\frac{2\alpha }{\beta }.$ Replacing the square root in Equation (10) with this approximation obtains

(11) $$\left\{\begin{array}{c} \left\{x,y\right\}=\left\{\left(\alpha +\frac{\beta }{4}\right)\;,\;\left(\alpha +\frac{\beta }{4}\right)\right\}\;\;\;\;\;\;\;\beta \leq 4\alpha \;\\ \left\{x,y\right\}\approx \left\{\left(\beta -\alpha \right)\;,\;\alpha \right\}\;\;\;\;\;\;\;\;\;\;\;\;\;\;\;\;\;\;\;\;\;\;\;\;\beta >4\alpha \end{array}\right.$$

The square root approximation used in Equation (11) is only reasonably accurate for small values of $\frac{4\alpha }{\beta }$, and becomes very inaccurate when $\frac{4\alpha }{\beta }$ takes on values near 1. The nature of this approximation has important consequences for model interpretation. A recent study [25] claimed that Bulmer and Parker’s model implies the following testable prediction: under anisogamy, the ratio of macrogamete to microgamete size (*x*/*y*) should be larger than three. This interpretation hinges on Equation (11), which suggests there is a discontinuity in the gamete size ratio as the threshold β=4α is passed and isogamy becomes unstable. The apparent discontinuity, however, is a consequence of the approximate nature of Equation (11) as can be seen by plotting gamete size ratios for the exact and approximate solutions as a function of $\frac{\beta }{\alpha }$. Equation (10) yields the following exact expression for the gamete size ratio:

(12) $$\left\{\begin{array}{c} \frac{x}{y}=1\;\;\;\;\;\;\;\;\;\;\;\;\;\;\;\;\;\;\;\;\;\;\;\;\;\;\;\;\;\;\;\;\;\;\;\;\;\;\;\;\;\;\;\;\;\;\;\;\;\;\;\;\;\;\;\;\;\;\;\;\;\;\;\;\beta \leq 4\alpha \;\\ \frac{x}{y}=\left(1+\sqrt{1-\frac{4\alpha }{\beta }}\right)/\left(1-\sqrt{1-\frac{4\alpha }{\beta }}\right)\;\;\;\;\;\;\;\;\;\;\beta \;>4\alpha \end{array}\right.$$

whereas Equation (11) obtains the approximate expression

(13) $$\left\{\begin{array}{c} \frac{x}{y}=1\;\;\;\;\;\;\;\;\;\;\;\;\;\;\;\;\;\beta \leq 4\alpha \;\\ \frac{x}{y}=\frac{\beta }{\alpha }-1\;\;\;\;\;\;\;\;\;\;\beta >4\alpha \end{array}\right.$$

**Figure 1:**
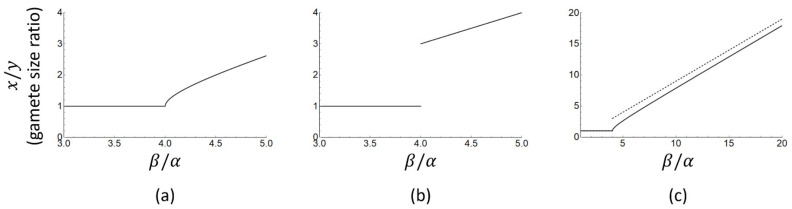
Exact (panel (**a**), Equation (12)) and approximate (panel (**b**), Equation (13)) solutions for the anisogamy ratio (ratio of macrogamete to microgamete size) as a function of $\frac{\beta }{\alpha }$, based on the model of Bulmer and Parker [15]. The discontinuity in panel (**b**) is not a prediction of the model as argued in [25], but an artefact arising from the approximation used in Equation (13). Panel (**c**) shows the exact (solid line) and approximate (dashed line) solutions over a larger range of $\frac{\beta }{\alpha }$, combined in a single panel.

The approximate Equation (13) does indeed suggest a discontinuity in gamete size ratio (*x*/*y,* Figure 1b) which jumps from 1 (isogamy) to 3 or more (anisogamy) as the ratio $\frac{\beta }{\alpha }$ increases. The exact analytical solution (Figure 1a), however, is continuous and the gamete size ratio can take on any value equal to or larger than one. The discontinuity in gamete size ratio in the approximate solution appears exactly where the approximation is the least accurate (i.e., around $\frac{4\alpha }{\beta }=1$). In summary, the exact and approximate solutions both predict that a switch from isogamy to anisogamy and a subsequent increase in gamete size ratio follow from an increase in the ratio $\frac{\beta }{\alpha }$, but the discontinuous jump in gamete size ratio as derived and tested in [25] is an artefact of approximate solution methods, not a model prediction.

### 2.2. Robustness of the Model to Assumptions on Zygote Survival, Gamete Survival, and Gamete Size-Number Trade-Offs

There are three mathematical functions at the core of the model presented in Section 2.1: $n\left(x\right)$, $g\left(x\right)$ and $f\left(x,y\right)$ (Equation (1)). These functions represent some of the assumptions built into the model, and it is important to consider the robustness of the model to alterations in these assumptions. In particular, there has been some controversy regarding the robustness of gamete competition models of anisogamy evolution to assumptions on zygote survival ($f\left(x,y\right)$). Randerson and Hurst [19] claimed that the theory can only explain the evolution of anisogamy if an unusual and untested assumption is made regarding the relationship between zygote size and zygote survival, namely that zygote fitness must increase disproportionately with its size at least over part of its size range [1,11,12]. This claim was debated in subsequent correspondence [20,21] and analysed in further detail in a game theoretical analysis of the model [15], in which the authors found that the theory is in fact quite robust to altered assumptions regarding the relationship between zygote size and survival. For example, survival functions that are approximately sigmoidal in shape (i.e., with an initial convex (‘accelerating’) phase, and a subsequent concave (‘decelerating’) phase for larger zygote sizes) can give rise to anisogamy [15]. Rather than being an unusual assumption, such a shape may arise in a natural way from simple biological considerations: a convex initial phase arises from the assumption that there is a minimum size below which zygotes cannot survive or have very low survival chances. A subsequent concave phase can arise from the fact that survival probability must eventually saturate as it certainly cannot reach values larger than 1, or from some other limit to the fitness of individual offspring that cannot be exceeded no matter how great the parental investment (see, e.g., [26] for verbal justification for such sigmoidal zygote size-survival relationships, and [21,27] for mathematical derivations). From a theoretical perspective, the eventual saturation of the function is not necessary for the evolution of anisogamy, such as in the models of Parker, Baker and Smith [1] and Charlesworth [12] which both examined zygote survival functions of the form $f\left(x,y\right)=c{\left(x+y\right)}^{h}$, where *c* is a constant of proportionality and the constant *h* determines the steepness of the curve. When *h* > 1, the resulting convex function generates anisogamy. Interestingly, although Parker, Baker and Smith [1] introduced this function as a simple intuitive first approximation with no particular biological grounding, it can in fact be derived from reasonable biological assumptions (derivation in Appendix A). Overall, contrary to needing unusual assumptions regarding zygote survival, the theory is relatively robust to these assumptions.

Similarly, various assumptions regarding the relationship between gamete size and gamete survival ($g\left(x\right)$), can also generate anisogamy [15,18]. Theory suggests that the size-dependence of zygote survival and the size-dependence of gamete survival, and the relationship of these two factors is a central determinant for when either isogamy or anisogamy is stable [15]. For example, in the model of Section 2.1 it was found that there is a switch from isogamy to anisogamy when the parameter β exceeds the value 4α, where β is the parameter that determines the strength of the zygote size-survival relationship, while α determines the strength of the gamete size-survival relationship (see, e.g., [15,18] for further details). Qualitatively similar results have been found, e.g., with the function $g\left(x\right)=e^{-\alpha /x}$ used in Section 2.1 [15], with stepwise gamete survival where all gametes above a certain threshold size survive [15], and with a dynamic approach to gamete survival where mortality rate of gametes is size-dependent and the actual probability of gamete survival until fertilisation depends on the time elapsed until a gamete of the opposite type is found [18].

Regarding the third component of Equation (1), $n\left(x\right)$, Parker (ref. [22], p. 36), suggested that the overall logic of the theory is likely not dependent on an exact trade-off between gamete size and number. To my knowledge, this conjecture has so far not been analysed mathematically. The function $n\left(x\right)=M/x$ used in Section 2.1 and in most models of the evolution of anisogamy to date can equivalently be written as x=M/n which simply means that the total resource *M* is converted into an arbitrarily large number of gametes with maximal efficiency: none of the resource *M* is lost, and the outcome is *n* gametes of size M/n, as if cutting a cake of size *M* into *n* pieces with perfect precision. *M* is typically considered a fixed energy budget which implies that the total resource cannot increase, but it is easy to imagine that the division process itself takes up some energy, so that some material is lost upon dividing the resource into smaller and smaller gametes. Perhaps the most obvious way to model such a scenario is to consider functions of the form

(14) $$x=M/n^{k}$$

where k≥1 is a constant, and values k>1 indicate some inefficiency in the division process. Such a function has a very natural biological interpretation which becomes transparent by switching to an alternative parametrisation. A simple way to divide the gametic resource into smaller gametes is by successive cell divisions [1,28]: *d* subsequent rounds of division into two equal parts will result in $n=2^{d}$ gametes. Rewriting and reparametrising the function $x=M/n^{k}$, we have $x=\frac{M}{n^{k}}=\frac{Mn^{1-k}}{n}=\frac{M{\left(2^{d}\right)}^{1-k}}{2^{d}}=\frac{M{\left(2^{1-k}\right)}^{d}}{2^{d}}=\frac{Mq^{d}}{2^{d}}$, where $\left(1-q\right)=\left(1-2^{1-k}\right)$ is the fraction of the gametic resource that is lost due to some inefficiency at each successive round of cell division. Thus, although Equation (14) is perhaps the simplest generalisation of the gamete size-number trade-off, the alternative parametrisation demonstrates that it has a concrete biological meaning. For the purposes of the current model we retain the form $x=M/n^{k}$, or equivalently

(15) $$n\left(x\right)={\left(\frac{M}{x}\right)}^{\frac{1}{k}}$$

Having established that Equation (15) is a reasonable expression for a generalised gamete size-number trade-off, we can substitute Equation (15) into Equation (1), while leaving $g\left(x\right)$ and $f\left(x,y\right)$ unchanged. Logarithmic fitness then becomes

(16) $$\left\{\begin{array}{c} \ln w_{x}=\ln{\left(\frac{M}{x}\right)}^{\frac{1}{k}}-\frac{\alpha }{x}-\frac{\beta }{x+y}=\frac{1}{k}\left[\ln\left(M\right)-\ln\left(x\right)-\frac{\alpha k}{x}-\frac{\beta k}{x+y}\right]\\ \ln w_{y}=\ln{\left(\frac{M}{x}\right)}^{\frac{1}{k}}-\frac{\alpha }{y}-\frac{\beta }{x+y}=\frac{1}{k}\left[\ln\left(M\right)-\ln\left(y\right)-\frac{\alpha k}{y}-\frac{\beta k}{x+y}\right] \end{array}\right.$$

and the logarithmic derivatives:

(17) $$\left\{\begin{array}{c} \frac{\partial \ln w_{x}}{\partial x}=\frac{1}{k}\left[\frac{\alpha k}{x^{2}}+\frac{\beta k}{{\left(x+y\right)}^{2}}-\frac{1}{x}\right]=0\\ \frac{\partial \ln w_{y}}{\partial y}=\frac{1}{k}\left[\frac{\alpha k}{y^{2}}+\frac{\beta k}{{\left(x+y\right)}^{2}}-\frac{1}{y}\right]=0 \end{array}\right.$$

The form of Equation (17) is familiar from Section 2.1: The component in square brackets is identical to Equation (3), but with α and β replaced by αk and βk, respectively. The parameter *k* is a constant, and therefore no further calculations are needed: the solution derived in Section 2.1 (Equation (10)) applies where we simply replace α and β by αk and βk.

(18) $$\left\{\begin{array}{c} \left\{x,y\right\}=\left\{k\left(\alpha +\frac{\beta }{4}\right)\;,\;\;k\left(\alpha +\frac{\beta }{4}\right)\right\}\;\;\;\;\;\;\;\;\;\;\;\;\;\;\;\;\;\;\;\;\;\;\;\;\;\;\;\;\;\;\;\;\;\;\;\;\;\;\;\;\;\beta \leq 4\alpha \;\\ \left\{x,y\right\}=\left\{k\frac{\beta }{2}\left(1+\sqrt{1-\frac{4\alpha }{\beta }}\right)\;,\;\;k\frac{\beta }{2}\left(1-\sqrt{1-\frac{4\alpha }{\beta }}\right)\right\}\;\;\;\;\;\;\;\;\;\;\;\beta >4\alpha \end{array}\right.$$

In other words, if the trade-off between gamete size and number is not exact and suffers from some inefficiency of the type in Equations (14) and (15), the equilibria are simply multiplied by the parameter *k*: since k>1 gamete size is increased at both isogamous and anisogamous equilibria in this model. The threshold at which the isogamous equilibrium is replaced by anisogamy (β>4α) is unchanged, as is the expression for the gamete size ratio (Equation (12)): in both cases the parameter *k* cancels out of the equation. Overall, the result confirms Parker’s [22] conjecture that qualitatively the theory is not dependent on an exact trade-off between gamete size and number, although quantitatively the results may differ.

An additional consequence of the above is that if the parameter *k* exceeds one (i.e., the gamete size-number trade-off is inefficient) zygote survival does not necessarily need to increase faster than linearly with zygote size. Recall that early work showed that a concave (accelerating) relationship between zygote size and survival is necessary for anisogamy to evolve [1,12]. A linear (or weaker) relationship between zygote size and survival, however, may be enough if producing large numbers of gametes is inefficient. This is easy to see by considering a simpler version of the model where, instead of having a separate function g for gamete survival, we assume a critical minimum size for viable gametes, and all gametes above this limit survive to mate if they find a partner gamete. This is similar to the “The threshold model for gametic survival” in Bulmer and Parker [15]. In this model, if the gametes are above the minimum size, the fitness functions of Equation (1) become

(19) $$\left\{\begin{array}{c} w_{x}\left(x,y\right)=n\left(x\right)f\left(x,y\right)\\ w_{y}\left(x,y\right)=n\left(y\right)f\left(x,y\right) \end{array}\right.$$

More specifically, it has been shown [1,12] that if there is an exact size-number trade-off $\left(n\left(x\right)=M/x\right)$ and if survival is of the form $f\left(x,y\right)=c{\left(x+y\right)}^{h}$, where c and h are constants, then anisogamy can evolve only if h>1. Logarithmic fitness derivatives under these assumptions are

(20) $$\left\{\begin{array}{c} \frac{\partial \ln w_{x}}{\partial x}=-\frac{1}{x}+\frac{h}{x+y}=0\\ \frac{\partial \ln w_{y}}{\partial y}=-\frac{1}{y}+\frac{h}{x+y}=0 \end{array}\right.$$

Now consider an otherwise similar model but with inexact gamete size-number trade-off as before $\left(n\left(x\right)={\left(\frac{M}{x}\right)}^{\frac{1}{k}}\right)$, and the corresponding logarithmic derivatives:

(21) $$\left\{\begin{array}{c} \frac{\partial \ln w_{x}}{\partial x}=\frac{1}{k}\left[-\frac{1}{x}+\frac{hk}{x+y}\right]=0\\ \frac{\partial \ln w_{y}}{\partial y}=\frac{1}{k}\left[-\frac{1}{y}+\frac{hk}{x+y}\right]=0 \end{array}\right.$$

Using logic analogous to the analysis of Equation (17), the components in square brackets in (21) are identical to Equation (20) with h replaced by hk. Given that Equation (20) is known to result in the evolution of anisogamy when h>1, the corresponding requirement for Equation (21) must be hk>1, or $h>\frac{1}{k}$. In other words, if the trade-off between gamete size and number is inefficient, the zygote size-survival relationship need not be convex: a linear, or even a concave zygote survival function can then suffice.

### 2.3. The Fisher Condition, A Paradox, and the Connection to Gamete Limitation

The two central drivers of gamete dimorphism in the disruptive selection theory of Parker, Baker and Smith [1] are gamete competition (i.e., maximizing the number of fertilisations in competition with others by increasing the number of gametes) and zygote provisioning (i.e., increasing the survival prospects of the zygote by increasing gamete size). Other selective pressures driving gamete size evolution have been proposed, and one of the most prominent, ‘gamete limitation’, is based on the idea that the fitness outcome from fertilisation does not only depend on competition between gametes from different individuals, but also on the overall fertilisation success. If fertilisation is very inefficient (for example, if the population is very sparse and gametes simply do not find each other) there may be selection to increase fertilisation prospects independent of any competition that may occur between gametes from different individuals. Consider a hypothetical situation where half of all eggs in a population are fertilized. Males may compete for these fertilised eggs, but they may also potentially increase their own fitness by fertilising a larger fraction of eggs. This is a simplified account of gamete limitation as a selective pressure for the evolution of anisogamy. Gamete limitation is the basis of the early group selection models of Kalmus [29] and Scudo [30], as well as some more recent models claiming that gamete competition is neither sufficient nor necessary for anisogamy [31] and that anisogamy did not arise from gamete competition and associated conflict, but as a tactic to maximize the contact rate between gametes in a broadcast spawning population [32,33].

How, then, does gamete limitation relate to the gamete competition models of PBS [1], Bulmer and Parker [15] and others? Are the two mutually exclusive explanations? Their connection becomes clear on examination of a seemingly paradoxical property of Bulmer and Parker’s model [15]. A closer look at Equation (1) reveals that the fitnesses of the two mating types are not necessarily equal, and the same issue is even clearer if we again use “The threshold model for gametic survival” (Equation (19)). From Equation (19) it is clear that $w_{x}$ and $w_{y}$ are equal only if $n\left(x\right)=n\left(y\right),\;$ which is generally the case only under isogamy. However, if exactly one *x*-gamete must fuse with exactly one *y*-gamete to form a zygote shared between the two parents, then the combined fitness of all *x* type individuals in a given period of time should be exactly equal to the combined fitness of all *y*-type individuals, regardless of asymmetries in gamete size and number [18]. This constraint is commonly called the ‘Fisher condition’, and it has important implications for evolutionary models [34]. If there are equally many adult individuals of each type (as there commonly are unless special conditions apply [35]), then the Fisher condition also implies that the average fitness of *x* and *y* individuals should be equal. Taken at face value, Equation (19) is clearly in conflict with this constraint. (Note, however, that this issue does not apply to the original PBS model [1] which did not make the assumption of pre-existing mating types, and instead assumed random fusion between all gametes).

A simple way to make Equation (19) ‘Fisher-consistent’ is to add a term *p* accounting for per-gamete fertilisation probability, which typically differs for the two gamete types:

(22) $$\left\{\begin{array}{c} w_{x}\left(x,y\right)=p_{x}n\left(x\right)f\left(x,y\right)\\ w_{y}\left(x,y\right)=p_{y}n\left(y\right)f\left(x,y\right) \end{array}\right.$$

Now, provided that $p_{x}n\left(x\right)=p_{y}n\left(y\right)$ the inconsistency mentioned above disappears. This raises a new question. A change in gamete numbers of either type may change fertilisation probabilities for both gamete types, so does the addition of the *p*-term change the model outcome? The answer is subtle and depends on population structure.

Consider first a very large panmictic population where gametes from all parents are randomly mixed. This implies that when a rare mutant producing a deviant number of gametes appears in the population, the change in mutant gamete number (diluted over the very large population) has a negligible effect on the per-gamete probability of fertilisation. Loosely speaking, under panmixia $p_{x}$ and $p_{y}$ are *not* functions of the mutant’s gamete size or number, so that when the fitness functions are differentiated for the mutant values *x* and *y*, the derivatives of $p_{x}$ and $p_{y}$ are both 0 and have no effect on the equilibria. Hence in a panmictic population the apparent incompatibility with the Fisher condition has no consequences for model results. Although Equations (1) and (19) taken at face value suggest that all gametes manage to be fertilised [33], in terms of the direction of selection and evolutionary outcome an equivalent interpretation is that gametes disperse and are well mixed in the population such that gamete competition is ubiquitous and that no focal individual has a significant impact on local fertilisation outcomes [18]. The random mixing of gametes over the population implies that gametes from each individual compete for fertilisations with gametes from all other individuals of the same type in the population, so gamete competition is in this sense at its strongest under panmixia.

If the population is not panmictic but instead gametes mix within local groups of relatively small size the situation is quite different. In a small group even a single mutant may have a significant effect on local gamete densities and hence fertilisation prospects [17,18,36]. Therefore, contrary to the panmictic case, under this kind of population structure $p_{x}$ and $p_{y}$ *are* functions of the mutant’s gamete output so that when fitness is differentiated for the mutant values of *x* and *y*, the derivatives of $p_{x}$ and $p_{y}$ differ from zero and potentially influence the equilibria. Under such group-structured conditions, gamete competition and gamete limitation can both select for increased gamete numbers simultaneously, and they tend to act in the same direction, reinforcing each other (see [8] and Figure 1 therein). Several mathematical models accounting for fertilisation success under various assumptions have been developed (reviewed in [37]) which can be used to model the components $p_{x}$ and $p_{y}$ and thus include selection via both gamete competition and gamete limitation in the same model.

An explicit consideration of the Fisher condition therefore exposes a very natural connection between ‘gamete competition’ and ‘gamete limitation’ models [18]. The combination of these model types has recently been termed ‘gamete dynamics’ theory [17,38]. A related and perhaps underappreciated fact is that gamete competition and gamete limitation are not mutually exclusive and can be present simultaneously. In a panmictic population each individual is effectively competing with all other individuals of the same type for fertilisations, but this does not rule out the possibility that the population is very sparse, gametes are bad at finding each other, or that fertilisation is otherwise very inefficient so that $p_{x}$ and $p_{y}$ in Equation (22) take on very small values. Arbitrarily high gamete limitation can therefore coexist with arbitrarily high gamete competition, but gamete limitation will tend to have a direct selective effect only in subdivided populations with local spawning groups. Even in populations with such group structure gamete competition is theoretically expected to dominate selection unless the spawning groups are very small [36]. However, at the extreme of just one individual of each mating type per spawning group there is no gamete competition, and in such a situation game limitation alone can drive the evolution of anisogamy [8,18]. In this conflict-free minimal group-size scenario the ideas presented in the early work of Kalmus [29] and Scudo [30] remain surprisingly valid, despite their group selection logic not holding in the more general case (see [18]).

The discussion in this section relates mainly to the effect that gamete density (gamete number per volume) has on fertilisation prospects. Aside from the density effect, a larger gamete is a larger target for other gametes and may consequently have better prospects of fertilisation than a smaller one (e.g., [39,40,41,42]). Classical gamete competition models or recent models combining gamete competition with gamete limitation [1,12,15,17,18] do not typically explicitly account for such gamete target size effects, and it may be of interest in future work to combine gamete competition, gamete limitation, and gamete size effects in a single model to allow easier comparison of different sources of selection side by side. None of these three selective effects (gamete competition, gamete limitation via gamete density, or gamete target size) are mutually exclusive, and a clear argument about their relative importance requires combining them in a single model in a consistent way.

## 3. Discussion

The PBS theory [1] is an explanation for one of the most consequential transitions in evolutionary history, shaping much of subsequent evolution and much of life as we now know it [2]. It is arguably the most widely accepted theory for this transition and for the evolutionary origin of the two sexes, with growing but as yet tentative empirical support [38,43,44,45,46]. The focus of this article has been not on the external empirical evidence, but on the internal robustness of the theory to various modifications of its assumptions.

There have of course been many earlier papers examining the robustness of the theory in various ways, or taking it in new directions. For example, Parker [47] showed that once evolved, anisogamy is extremely stable even if sperm competition later decreases, e.g., due to internal fertilisation. On the other hand, the logic of the theory works even in the (perhaps unlikely) case that internal fertilization evolved prior to anisogamy [17]. One centrally important paper is Brian Charlesworth’s population genetic model which showed that the PBS theory is robust to the existence of mating types: the theory works regardless of whether mating types preceded anisogamy or vice versa [12]. The original PBS model [1] was based on the assumption of random fusion between all gametes, irrespective of their size in a large, synchronously spawning population. However, mating types may have evolved prior to anisogamy [40]. Mating types are gamete genotypes for molecular mechanisms that regulate compatibility between fusing gametes so that only opposite types (e.g., *x* and *y* types in the models in this article, or + and − types in commonly used notation) can fuse with each other to form a zygote. If mating types did indeed evolve prior to anisogamy, then fusion would not have been random between all gametes, contradicting an assumption of the PBS model. Charlesworth [12] showed that a mutation changing gamete size may be in close linkage with the mating type locus, which will immediately lead to disassortative fusion for gamete size; or linkage between the gamete size mutation and mating type locus may initially be loose, in which case selection favours tighter linkage over time. In both cases the long-term evolutionary outcome is anisogamy with disassortative fusion for gamete size. Parker [10] presented a model for the evolution of disassortative fusions between large and small gametes during the evolution of anisogamy in the absence of pre-existing mating types. Most later models of the evolution of anisogamy, and all the models presented in this paper have assumed that mating types preceded anisogamy.

In this article we have seen that the PBS theory (in the game-theoretical form of [15]) is quite robust to several further alterations in its central assumptions. By robustness in this context I mean that the theory works even if the central functions that make up the model (*n*, *g*, *f* in Equation (1)) are altered in various ways, while remaining within some reasonable bounds. This kind of robustness could be considered a double-edged sword: It is a necessary property for the model to be plausible. If the theory was not robust in this sense and instead relied on a very specific shape for each of the central functions, we could have little faith in it because we can be almost certain that the specific shape is not exactly true in nature. The flipside of this is that because the theory does indeed work with a range of core functions, and because we do not know which one of them is correct, the model predictions cannot be considered accurate in a quantitative sense. For example, a change in the survival function *f* will likely result in a change in, e.g., the gamete size ratio (Equation (12)), or in the switch point from isogamy to anisogamy. For these reasons the PBS theory and its corollaries are amenable to comparative tests of predicted correlations (e.g., [38,43,44,45]), but much less so to qualitative tests of exact numerical predictions.

This is not a weakness of the theory. It is a consequence of the nature of the problem where some of the central components are perhaps forever unknowable in detail in the ancestral organisms where the transition took place. At least for now, we can only know very general properties that relationships between, e.g., gamete size and number, or zygote size and survival are likely to have. In such a situation the best possible modelling outcome is one where the model works within a wide range of plausible settings, resulting in possibly quantitatively different, but quantitative similar outcomes.

## Figures and Tables

**Table 1:** Glossary.

Term	Definition
Anisogamy	Size dimorphism of gametes: in an anisogamous system one gamete type is larger (e.g., ova) than the other (e.g., spermatozoa).
Female	Biologically, the female sex is defined as the adult phenotype that produces the larger gametes in anisogamous systems.
Isogamy	A gametic system where all gametes are of similar size.
Male	Biologically, the male sex is defined as the adult phenotype that produces the smaller gametes in anisogamous systems.
Mating types	Mating types are gamete genotypes for molecular mechanisms that regulate compatibility between fusing gametes. Mating types guarantee disassortative fusion.

**Figure A1:**
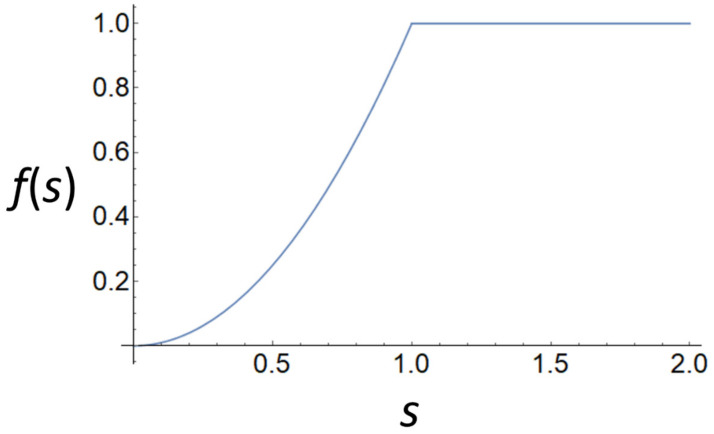
Equation (A8) with parameter values *c* = 1 and *h* = 2.

## Appendix A. Derivation of the Parker, Baker and Smith 1972 Zygote Survival Function

Some early models of the evolution of anisogamy (e.g., [1,12]) modelled the relationship between zygote size and zygote survival with a power function of the form

(A1) $$f\left(s\right)=cs^{h}$$

where *c* and *h* are positive parameters, and *s* is zygote size, so that in the notation of models in the main text s=x+y.

PBS [1] introduced this function as a simple and intuitive first approximation that is flexible enough to model linear, decelerating, and accelerating relationships but not based on biological justification. Here I show that Equation (A1) does arise from simple biological assumptions. The underlying principle is similar to that in [19,21,27,48]: assume that there is constant mortality μ during growth to maturity, and that growth time is a function *T*(*s*) of initial zygote size *s*. I initially assume that time to maturity *T* is composed only of the growth time from initial size *s* to size at maturity *b* (but see Equations (A9)–(A11)).

Instead of assuming a given shape for *T*(*s*) as in in [19,21,27,48], I take a step back and assume that growth *rate* at time *t* is proportional to current size *z*(*t*) (where *z*(0) = *s* and *z*(*T*) = *b*). This is a special case of growth rate as a power function (Equation (3) in [49], argued to be a reasonable description of prematurity growth) with exponent 1. The claim here is not that the specific exponent 1 is likely to be exactly true in nature: Day & Taylor [49], for example, use an exponent of 2/3. Instead, the point is that the zygote survival function of early models [1,12] is grounded in published biological models and principles. Under these assumptions, growth is then described by the following equation:

(A2) $$\frac{dz\left(t\right)}{dt}=az\left(t\right)$$

where *a* is a constant (growth rate parameter [49]). (A2) is a differential equation with initial value *z*(0) = *s* and straightforward to solve:

(A3) $$\frac{dz}{dt}=az\;\to \;dz/z=adt\;\to \;\int \frac{1}{z}dz=\int adt\;\to \;\ln\left(z\right)=at+C$$

Now use the initial value $z\left(0\right)=s$ to compute the value of the integration constant:

$\ln\left(s\right)=a*0+C$→ $C=\ln\left(s\right)$ which we can plug back into Equation (A3):

$\ln\left(z\right)=at+\ln\left(s\right)$, or $t=\frac{\ln\left(z\right)-\ln\left(s\right)}{a}$

In other words, it takes time $t=\frac{\ln\left(z\right)-\ln\left(s\right)}{a}$ to grow from initial size *s* to size *z*. Additionally, given that *z*(*T*) = *b*, the time to grow to mature size is

(A4) $$T=\frac{ln\left[z\left(T\right)\right]-ln\left[s\right]}{a}=\frac{ln\left[b\right]-ln\left[s\right]}{a}=\frac{1}{a}\ln\left(\frac{b}{s}\right)$$

Constant mortality μ during growth implies that survival to age *T* is:

(A5) $$f\left(s\right)=e^{-\mu T}=e^{-\mu \frac{1}{a}\ln\left(\frac{b}{s}\right)}={\left(e^{\ln\left(\frac{b}{s}\right)}\right)}^{-\frac{\mu }{a}}={\left(\frac{b}{s}\right)}^{-\frac{\mu }{a}}={\left(\frac{s}{b}\right)}^{\frac{\mu }{a}}=b^{\left(-\frac{\mu }{a}\right)}s^{\frac{\mu }{a}}$$

*b*, *a* and μ are constants, so we can reparameterise as $c=b^{\left(-\frac{\mu }{a}\right)}$ and $h=\frac{\mu }{a}$, resulting in

(A6) $$f\left(s\right)=cs^{h}$$

which is the power function used in [1,12].

Of course, growth time cannot be negative, so Equation (A4) should be amended:

(A7) $$T=\left\{\begin{array}{c} \frac{1}{a}\ln\left(\frac{b}{s}\right)\;\;\;\;\;s<b\\ 0\;\;\;\;\;\;\;\;\;\;\;\;\;\;\;\;\;s\geq b \end{array}\right.$$

Consequently Equation (A6) becomes

(A8) $$f\left(s\right)=\min\left(1,cs^{h}\right)$$

which is approximately sigmoidal if *h* > *1* (see Figure A1 for an example). The requirement h>1 is equivalent to $\frac{\mu }{a}>1$ or μ>a (mortality rate is higher than the growth rate parameter).

Note that the general shape of the curve would not change if the function *T* included an additional constant (as in [19,21,27,48]):

(A9) $$T=\frac{1}{a}\ln\left(\frac{b}{s}\right)+v$$

Then

(A10) $$f\left(s\right)=e^{-\mu T}=e^{-\mu v-\mu \left(\frac{1}{a}\ln\left(\frac{b}{s}\right)\right)}=e^{-\mu v}e^{-\mu \left(\frac{1}{a}\ln\left(\frac{b}{s}\right)\right)}=e^{-\mu v}b^{\left(-\frac{\mu }{a}\right)}s^{\frac{\mu }{a}}$$

Now if we write $c=e^{-\mu v}b^{\left(-\frac{\mu }{a}\right)}$ and $h=\frac{\mu }{a}$ we have again

(A11) $$f\left(s\right)=cs^{h}$$

where *c* and *h* are constants.
